# Supplementary material for: A Critical View of the Application of the APEX Software (Aqueous Photochemistry of Environmentally-Occurring Xenobiotics) to Predict Photoreaction Kinetics in Surface Freshwaters
Source: Molecules. 2019 Dec 18;25(1):9. doi: 10.3390/molecules25010009 (PMC7017383; doi:10.3390/molecules25010009)
Supplement: Supplementary file 1 [file molecules-25-00009-s001.zip › 3.2.4_gcc-4.4.0.zip!/3.2.4_gcc-4.4.0/3.2.4_gcc-4.4.0/doc/octave/html/interpreter/Information.html]

Information - Untitled


Next: Operators and Functions,
Previous: Creating Sparse Matrices,
Up: Basics


---

#### 21.1.3 Finding out Information about Sparse Matrices

There are a number of functions that allow information concerning
sparse matrices to be obtained. The most basic of these is
issparse that identifies whether a particular Octave object is
in fact a sparse matrix.

Another very basic function is nnz that returns the number of
non-zero entries there are in a sparse matrix, while the function
nzmax returns the amount of storage allocated to the sparse
matrix. Note that Octave tends to crop unused memory at the first
opportunity for sparse objects. There are some cases of user created
sparse objects where the value returned by nzmax will not be
the same as nnz, but in general they will give the same
result. The function spstats returns some basic statistics on
the columns of a sparse matrix including the number of elements, the
mean and the variance of each column.

— Loadable Function: **issparse** (expr)  
> Return 1 if the value of the expression expr is a sparse matrix.

— Built-in Function: scalar = **nnz** (a)  
> Returns the number of non zero elements in a.
>
>
>
> **See also:** sparse.

— Function File: **nonzeros** (s)  
> Returns a vector of the non-zero values of the sparse matrix s.

— Built-in Function: scalar = **nzmax** (SM)  
> Return the amount of storage allocated to the sparse matrix SM.
> Note that Octave tends to crop unused memory at the first opportunity
> for sparse objects. There are some cases of user created sparse objects
> where the value returned by nzmax will not be the same as nnz,
> but in general they will give the same result.
>
>
>
> **See also:** sparse, spalloc.

— Function File: [count, mean, var] = **spstats** (s)  
— Function File: [count, mean, var] = **spstats** (s, j)  
> Return the stats for the non-zero elements of the sparse matrix s.
> count is the number of non-zeros in each column, mean
> is the mean of the non-zeros in each column, and var is the
> variance of the non-zeros in each column.
>
> Called with two input arguments, if s is the data and j
> is the bin number for the data, compute the stats for each bin. In
> this case, bins can contain data values of zero, whereas with
> `spstats (`s`)` the zeros may disappear.

When solving linear equations involving sparse matrices Octave
determines the means to solve the equation based on the type of the
matrix as discussed in Sparse Linear Algebra. Octave probes the
matrix type when the div (/) or ldiv (\) operator is first used with
the matrix and then caches the type. However the matrix\_type
function can be used to determine the type of the sparse matrix prior
to use of the div or ldiv operators. For example

```
     a = tril (sprandn(1024, 1024, 0.02), -1) ...
         + speye(1024);
     matrix_type (a);
     ans = Lower
```

show that Octave correctly determines the matrix type for lower
triangular matrices. matrix\_type can also be used to force
the type of a matrix to be a particular type. For example

```
     a = matrix_type (tril (sprandn (1024, ...
        1024, 0.02), -1) + speye(1024), 'Lower');
```

This allows the cost of determining the matrix type to be
avoided. However, incorrectly defining the matrix type will result in
incorrect results from solutions of linear equations, and so it is
entirely the responsibility of the user to correctly identify the
matrix type

There are several graphical means of finding out information about
sparse matrices. The first is the spy command, which displays
the structure of the non-zero elements of the
matrix. See fig:spmatrix, for an example of the use of
spy. More advanced graphical information can be obtained with the
treeplot, etreeplot and gplot commands.

**Figure 21.1: Structure of simple sparse matrix.**

One use of sparse matrices is in graph theory, where the
interconnections between nodes are represented as an adjacency
matrix. That is, if the i-th node in a graph is connected to the j-th
node. Then the ij-th node (and in the case of undirected graphs the
ji-th node) of the sparse adjacency matrix is non-zero. If each node
is then associated with a set of coordinates, then the gplot
command can be used to graphically display the interconnections
between nodes.

As a trivial example of the use of gplot, consider the example

```
     A = sparse([2,6,1,3,2,4,3,5,4,6,1,5],
         [1,1,2,2,3,3,4,4,5,5,6,6],1,6,6);
     xy = [0,4,8,6,4,2;5,0,5,7,5,7]';
     gplot(A,xy)
```

which creates an adjacency matrix `A` where node 1 is connected
to nodes 2 and 6, node 2 with nodes 1 and 3, etc. The coordinates of
the nodes are given in the n-by-2 matrix `xy`.
See fig:gplot.

**Figure 21.2: Simple use of the gplot command.**

The dependencies between the nodes of a Cholesky factorization can be
calculated in linear time without explicitly needing to calculate the
Cholesky factorization by the `etree` command. This command
returns the elimination tree of the matrix and can be displayed
graphically by the command `treeplot(etree(A))` if `A` is
symmetric or `treeplot(etree(A+A'))` otherwise.

— Function File: **spy** (x)  
— Function File: **spy** (..., markersize)  
— Function File: **spy** (..., line\_spec)  
> Plot the sparsity pattern of the sparse matrix x. If the argument
> markersize is given as an scalar value, it is used to determine the
> point size in the plot. If the string line\_spec is given it is
> passed to `plot` and determines the appearance of the plot.
>
>
>
> **See also:** plot.

— Loadable Function: p = **etree** (s)  
— Loadable Function: p = **etree** (s, typ)  
— Loadable Function: [p, q] = **etree** (s, typ)  
> Returns the elimination tree for the matrix s. By default s
> is assumed to be symmetric and the symmetric elimination tree is
> returned. The argument typ controls whether a symmetric or
> column elimination tree is returned. Valid values of typ are
> 'sym' or 'col', for symmetric or column elimination tree respectively
>
> Called with a second argument, etree also returns the postorder
> permutations on the tree.

— Function File: **etreeplot** (tree)  
— Function File: **etreeplot** (tree, node\_style, edge\_style)  
> Plot the elimination tree of the matrix s or
> s`+`s`'` if s in non-symmetric. The optional
> parameters line\_style and edge\_style define the output
> style.
>
>
>
> **See also:** treeplot, gplot.

— Function File: **gplot** (a, xy)  
— Function File: **gplot** (a, xy, line\_style)  
— Function File: [x, y] = **gplot** (a, xy)  
> Plot a graph defined by A and xy in the graph theory
> sense. A is the adjacency matrix of the array to be plotted
> and xy is an n-by-2 matrix containing the coordinates of
> the nodes of the graph.
>
> The optional parameter line\_style defines the output style for
> the plot. Called with no output arguments the graph is plotted
> directly. Otherwise, return the coordinates of the plot in x
> and y.
>
>
>
> **See also:** treeplot, etreeplot, spy.

— Function File: **treeplot** (tree)  
— Function File: **treeplot** (tree, line\_style, edge\_style)  
> Produces a graph of tree or forest. The first argument is vector of
> predecessors, optional parameters line\_style and edge\_style
> define the output style. The complexity of the algorithm is O(n) in
> terms of is time and memory requirements.
>
>
>
> **See also:** etreeplot, gplot.

— Function File: **treelayout** (Tree)  
— Function File: **treelayout** (Tree, permutation)  
> treelayout lays out a tree or a forest. The first argument Tree is a vector of
> predecessors, optional parameter permutation is an optional postorder permutation.
> The complexity of the algorithm is O(n) in
> terms of time and memory requirements.
>
>
>
> **See also:** etreeplot, gplot, treeplot.
